# Spread of infectious agents through the air in complex spaces

**DOI:** 10.1098/rsfs.2021.0080

**Published:** 2022-02-11

**Authors:** Ian Eames, Jan-Bert Flór

**Affiliations:** ^1^ Centre for Engineering in Extreme Environments, University College London, Gower Street, London WC1E 7JE, UK; ^2^ Laboratoire des Écoulements Géophysiques et Industriels (LEGI), CNRS, Université Grenoble Alpes, Grenoble INP, Grenoble 38000, France

**Keywords:** COVID-19, airborne infection, turbulence, dispersion

## Abstract

The fluid mechanical processes that govern the spread of infectious agents through the air in complex spaces are reviewed and the scientific gaps and challenges identified and discussed. Air, expelled from the nose and mouth, creates turbulent jets that form loosely coherent structures which quickly slow. For the transport and dispersion of aerosols, the suitability of the Eulerian as well as the Lagrangian approaches are brought into context. The effects of buoyancy and external turbulence are explored and shown to influence the horizontal extent of expulsion through distinct mechanisms which both inhibit penetration and enhance mixing. The general influence of inhomogeneous turbulence and stratification on the spread of infectious agents in enclosed complex spaces is discussed.

## 1. Introduction

The consequence of dealing with COVID-19 has impacted lives and the way we lead them [1]. The disruption caused by COVID-19 has highlighted the importance of understanding how infectious diseases spread through the air. Airborne diseases and limiting their spread has been a core area of activity for virologists, microbiologists and environmental engineers. Significant advances were made during the SARS epidemic of 2009, particularly in the adoption and application of new methodologies to assess transport mechanisms and this has provided a platform to take COVID-19 research forward.

A large component of the transmission route (the generation of infectious agents and their movement through and by the air) involves a core aspect of engineering—fluid mechanics. Transmission is complex owing to the necessity of dealing with the ‘lived’ environment, but to solve practical problems requires a level of abstraction. The major challenge from a research perspective is to identify which key physical processes need to be retained in an abstracted model that are crucial to the real world setting. For this reason, we start with a statement of the scope and complexity of the problem of the spread of infectious agents in complex spaces.

## 2. Complex spaces and environments

Many diseases (such as SARS, influenza, varicella, tuberculosis) are transmitted in part, or wholly, through the air, with the dispersal occurring due to the movement of aerosols, particles or droplets that carry bacteria and viruses [2]. Dispersed matter can enter the air through fragmenting mucus strands deep in the lung, air shear stress removing liquid film within the respiratory tract, invasive medical AGPs, across to resuspension from activities such as walking, bed making and cleaning. The risk of transmission may come from inhalation, ingestion or contact through the skin or eyes.

There is now greater recognition of the importance of bringing ‘clean’ air into living and working spaces. The two main ways to accomplish this are through either mechanical ventilation, which employs a fan to drive air into and out from a room or natural ventilation, which uses buoyancy/temperature differences to drive an exchange between spaces. These two flushing techniques are quite distinct: mechanical ventilation uses inhomogeneous turbulence to dilute contaminants and a mean flow to flush through the air, while natural ventilation uses buoyancy and entrainment to drive an exchange flow. Both the level of turbulence and density contrast vary significantly over these spaces and with these two modes of ventilation.

The conveyance of individuals by bus, train, tube or plane usually involves high occupancy levels, which has led to superspreading events (e.g [3,4]) due to cross infection. Some trains still permit limited control of local environment with small windows, whereas the faster TGV trains are ventilated in a way similar to planes. The ambient air tends to be introduced under mechanical ventilation, with fresh air thermally conditioned before being introduced into the carriage. Aircraft are a special case because of the high density of people in a closed environment, requiring high mechanical ventilation with HEPA scrubbers to remove aerosols, thermal conditioning to reduce relative humidity (RH) (to about 15%) and maintain a constant temperature (approx. 26°C). Stratification effects are generally weak within aircraft, except during embarkation and disembarkation, when only air-conditioning is in operation and clean air exchange occurs through open cabin doors. This poor air exchange is evident from the CO_2_ levels that typically rise to approximately 2000–2500 ppm and fall back to approximately 1600 ppm during travel. The close packing of passengers and seats significantly diminishes the capacity to mix and dilute contaminants in the air and leads to recirculating regions with high residence times.

Most homes are naturally ventilated with air heated by radiators, underfloor heating or fires, creating a temperature stratification that greatly influences mixing and dispersion. Clean air from outside is introduced by an exchange flow through vents, typically at contrasting heights. This type of exchange is potentially efficient at providing clean air—the low levels of turbulence means that small droplets simply sediment to the ground and aerosols are swept out usually through entrainment into rising plumes [5]. The drawback is that thermal stratification can limit the vertical exchange and dilution.

These examples highlight the importance of understanding the influence of turbulence and stratification on how matter (CO_2_, droplets, aerosols), expelled during breathing, is redistributed. In identifying research gaps, a great deal has been completed on the near-field effect of expulsion during breathing, focusing on the momentum dominated motion with limited attention to the initial effects of buoyancy. The conceptual picture of far field transport is reasonably clear except from the influence of turbulence on stratification in these complex configurations. So while the picture of near and far field is reasonably complete, a major gap remains in the link between these two fields, a process generally mediated by the action of buoyancy and turbulence. This provides a central theme to this paper.

## 3. Aerosol, droplets and viral load

### 3.1. Composition of breath

At sea level, clean air is composed of 79% nitrogen, 20.95% oxygen, 1% argon. While the atmospheric level of CO_2_ measured at the Mauna Loa Observatory is currently about 414.19 ppm (25 October 2021), the proximity to urban areas and local weather conditions both affect the background level of CO_2_, and can vary from 390 to 560 ppm. Exhaled breath contains a greater quantity of CO_2_ (typically 4–5% by volume) which can build up in enclosed spaces. The temperature of exhaled air is higher than the ambient air, and since the molecular mass of water is lower than nitrogen, RH decreases air density. RH is expressed as a fraction of the total saturation of water vapour, which is temperature dependent; the mass loading of vapour at saturation changes increases 0.023 kg m^−3^ (at 25°C) to 0.038 kg m^−3^ (at 35°C). At 25°C, the diffusivity of scalar species (such as temperature, CO_2_) are similar to the kinematic viscosity *ν* = *μ*_*a*_*ρ*_*a*_ = 1.5 × 10^−5^ m^2^ s^−1^, as anticipated from the kinetic theory of gases. The air density is *ρ*_*a*_ = 1.184 kg m^−3^. Transport of CO_2_ is a potentially useful metric for air quality notwithstanding the difficulty interpreting time series. Exhaled air also contains droplets and aerosols, which are characterized by a range of sizes, extending from submicrometre to millimetric scales, depending on the availability of saliva/mucus and the manner in which it enters the air.

### 3.2. Dispersed phase dynamics

The dynamics and characteristics of exhaled droplets—discrete elements largely composed of water—change due to evaporation or condensation depending on the RH and temperature [6]. Evaporation leads to a reduction in diameter and ultimately a rigid surface as residual mass is left by water loss. Despite this transition from droplets to (SARS-COV-2) aerosol particles, we keep the terminology of droplets for simplicity in this discussion. The movement of discrete droplets may be studied by (a) following them individually with time (a Lagrangian approach), which is useful for numbers of particles less than 10^6^, depending on the complexity of the equation of motion, or (b) analysing their average concentrations (an Eulerian approach). The benefit of the Lagrangian approach is that it can deal with multiple droplet sizes simultaneously and is capable of particle-crossing effects. However, it suffers from having poor statistical convergence when sampling at low particle concentrations.

Exhaled droplets (mass *m* and velocity ***v***) move through a turbulent flow generated during exhalation with an equation of motion of the form

3.1 $$\frac{d}{dt}(mv)=F_{T}+F_{D}+F_{G},$$

based on the action of a series of forces, i.e. on evaporation (***F***_*T*_), viscous and form drag (***F***_*D*_) and weight ($F_{G}=-mg\hat{z}$, *g* = 9.81 m s^−2^). This simplification of the equation of motion lies in the fact that the droplet density, *ρ*_*p*_, is larger than that of the air, *ρ*_*f*_. The loss of droplet mass due to evaporation generates a ‘thrust’ force, whose origin is the same as that from a rocket, except the evaporative flux relative to the droplet is zero, giving $F_{T}=\dot{m}v$. The drag force is semi-empirical and of the form $F_{D}=\frac{1}{8}\rho_{f}\pi d^{2}C_{D}\;|\;u-v\;|\;(u-v)$; the drag coefficient *C*_*D*_(*Re*_*p*_) is a function of the Reynolds number based on the local slip velocity, *Re*_*p*_ = |***u*** − ***v***|*d*/*ν*. *C*_*d*_ = (24/*Re*)(1 + 0.15*Re*^0.687^) (for 1 < *Re* < 1000) [7]. For small droplets, the equation of motion becomes linear,

3.2 $$\frac{dv}{dt}=\frac{1}{t_{d}}(u-v-v_{T}\hat{z}),$$

where the response time is *t*_*d*_ = *d*^2^*ρ*_*p*_/18*μ* is based on a droplet with a contaminated surface. The linear drag model is strictly valid for *d* ≤ 200 μm, for which the Reynolds number, based on the slip-velocity, is less than *μ* = 1.8 × 10^−5^ Pa at 18°C. Since the liquid droplets are much more viscous than air and usually contaminated (with pathogens, blood corpuscles and salt), the estimate of *t*_*p*_ is appropriate. Droplets have a slip velocity relative to the flow, giving for the non-evaporating case, *d* = 1–10 μm, fall velocities varying from 0.5 to 5 mm s^−1^. When droplets are small, they have a tendency to follow the flow, with the effect of small inertia leading them being expelled from vortical regions. The ability of a droplet to respond to an evolving flow is characterized by the Stokes number, *St* = *ut*_*p*_/*L* (where *u*, *L* are the characteristic flow speed and lengthscale of the flow, respectively). When *St* ≪ 1, droplets tend to follow the flow, while when *St* ≫ 1, the droplet pasts through the flow and the motion is ballistic.

A critical component of droplet dynamics is their loss of mass due to evaporation. Humidity and temperature may change the droplet size, with the potential of enhancing or suppressing sedimentation. The reduction in droplet diameter generally follows a *d*^2^ law, where the rate of decrease of *d*^2^ is typically a constant d*d*^2^/d*t* = −*D*_*f*_ [8], when the diameter is above a critical value *d* > *d*_min_. The reduction in the droplet diameter, parametrized as a diffusivity (*D*_*f*_), depends on relative humidity, the droplet temperature and ambient temperature. The turbulence and stratification determine their residence time in the air during which this reduction may take place. Below, first the droplet and aerosol characteristics are discussed in relation to the viral load, and the turbulence and stratification aspects in §§4 and 5 thereafter.

### 3.3. Aerosol/droplet characteristics and infectivity

When a person sneezes or coughs, the large expelled droplets of size (of about 100 μm) are carried more than 6 m by exhaled air at a velocity of 50 m s^−1^ in the case of sneezing, more than 2 m at a velocity of 10 m s^−1^ in the case of coughing, and less than 1 m at a velocity of 1 m s^−1^ in the case of breathing [9–12]. Aerosols are emitted in large numbers (typically approx. 46 000 particles per cough [13]), with a large variation depending on the person and speaking or breathing activity, and have the greatest potential to contribute to contamination, due to their large number, ease of dispersion, and ability to penetrate deep into the respiratory tracts of a human body [14].

**Figure 1.**
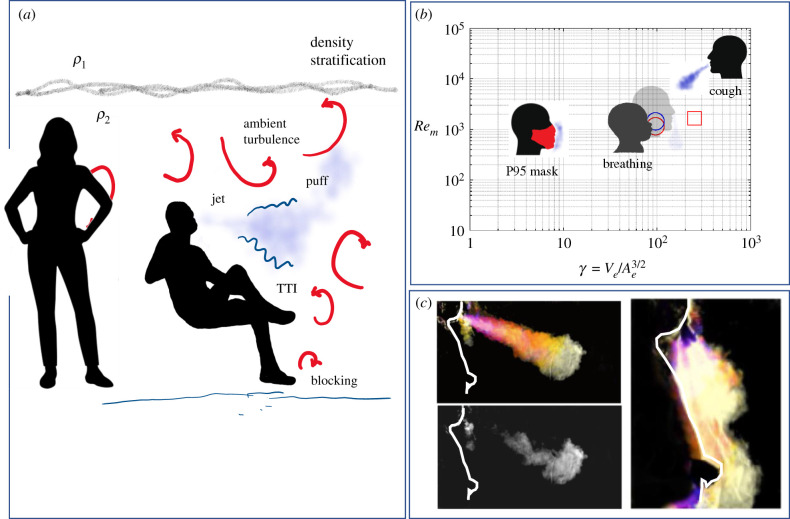
(*a*) Schematic showing environmental effects (turbulence and buoyancy) on the local dispersal of exhalation discharges. The jet exhaled into the turbulent environment creates a turbulent–turbulent interface (TTI). The turbulence interacts with a stratification, here represented as an interface between regions of density *ρ*_1_ and *ρ*_2_ ( > *ρ*_1_). (*b*) Regime diagram showing the parametric variation of the maximum Reynolds number *Re*_*m*_ as a function of $V_{e}/A_{e}^{3/2}$ for exhalation, where *V*_*e*_ is the volume exhaled through and area *A*_*e*_. The general position for breathing, coughing and tightly fitted mask are indicated, using the values suggested by Gupta *et al*. [16]. (*c*) Photographs of a smoke cloud being expelled from the nose and mouth, generating a downward and approximately horizontal jet, respectively.

Many experimental studies use bright lights to illuminate large expelled droplets (see e.g. [15], and references therein), which can be tracked in time and are observed to fall ‘out’ of the air in less than a minute. Small droplets and aerosols are too small to be individually identified and tracked in whole field views. Techniques that involve smoke are useful to determine collective movement and entrainment characteristics of expelled air (figure 1*c*). The presence of small droplets and aerosols is usually analysed using volume sampling with laser scattering (e.g. [17]). Aerosols and fine droplets are difficult to track over time and other techniques, such as (3D)PIV with Lagrangian diagnostics have been applied. The fluid mechanics community have a history of using analogue studies (e.g. [18]) to understand general physical processes.

Computational studies that track both the flow and particles seem to be the most instructive for unpicking complex processes. Chong *et al.* [19] analysed computationally the response of droplets to humidity and temperature in breath and showed that small droplets approximately 10 μm may initially grow as expelled air becomes saturated due a reduction in temperature. At later time, droplets shrink as they enter less humid environments [20], shrinking typically to approximately 10% of their initial diameter and consequently remaining longer in the air. About 85% of emitted viruses are in this smaller particle range [21].

The viral load of SARS-CoV-2 in saliva droplets, emitted with aerosols from coughs or sneezes, range from *N*_copies_ = 10^4^ to 10^8^ virus copies per millilitre, respectively [22–24]. The most infectious droplet class may be estimated from a simple statistical argument: the number of viral copies in a droplet of diameter *d* is *πd*^3^ *N*_copies_/6 and so the probability of infection from droplets whose size is described by a pdf *p*(*d*) is *p*_*I*_ = *p*(*d*)*πd*^3^ *N*_copies_/6. With a prescribed size distribution, the most infectious droplet size can be estimated. For example, for a lognormal size distribution with shape factor *σ* = 0.7, the most infectious droplet size is approximately 4.3*d*_*o*_ where *d*_*o*_ is the most common droplet size. Accounting for evaporation, the most infectious droplets far from the point of ejection are 4.3*α*^1/3^*d*_*o*_, where α∼1% is the solid fraction of within saliva. Here the most infectious droplet size is approximately 0.86 *d*_*o*_. For example, from a coughing study where *d*_*o*_ = 10 μm [25], the most infectious size within the room is 8 μm. To estimate the potential for infectivity requires knowledge of the viral load within the infectious fluid. A droplet of 10 μm, emitted by a sneeze, of 10 μm has a volume of 5 × 10^−10^ ml so that the average number of viral copies is approximately 10^−2^. Against this low probability of individual droplets containing a viral copy, is the potentially large number of droplets created, making the transport of viral loads in aerosols a matter of debate [22].

Analysis of the infectious rates across countries by Karim *et al*. [27] and Mecenas *et al*. [26] suggest that the infectious agents spread more readily in low humidity and low temperature regions. These macroscopic observations are consistent with the enhanced droplet residence time in the air due to evaporation in a low humidity environment. During the COVID-19 pandemic, research has been able to differentiated the influence of speech patterns and breathing modes on virus emission rate (e.g. [14]), and the effect of humidity, temperature, UV radiation and the ambient flow conditions of virus viability (see reviews of [15,28]).

## 4. Pneumodynamics

### 4.1. Breathing characteristics

A critical pathway for the spread of airborne infection is through exhalation. The aerodynamics of the human breath, or pneumodynamics, have an inherent complexity associated with compositional variation, turbulence and their interaction with the external environment. The movement of the ribcage and diaphragm lead to a volumetric change in the lungs. As a consequence an airflow is generated, with a density difference Δρ from the ambient, which passes through the upper-respiratory tract, exiting through an area *A*_*e*_. Under normal breathing conditions, air is exchanged from the lungs (capacity 6 l) periodically with a tidal volume of typically *V*_*e*_ = 0.5 l with exhalation typically over *t*_*e*_ ∼ 1.5 − 2.5 s. For processes that involve coughing, a number of studies have reported the volume flow rate, indicating a larger volume *V*_*e*_ ∼ 0.5 − 1.5 l expelled over a period *t*_*e*_ ∼ 0.4 s [16]. Sequential coughs occur typically in multiple expulsion volumes with decreasing magnitude. Coughing is with the mouth open and a variety of researchers have estimated areas *A*_*e*_ ∼ 1–4 cm^2^; through the nose, expulsion area ranges from *A*_*e*_ ∼ 1 to 2 cm^2^. The direction of the expelled air depends on the orientation of the head and whether expulsion is through nose or mouth.

The dimensionless groups formed from *V*_*e*_, *A*_*e*_, *t*_*e*_, Δ*ρ* are the geometrical measure *γ*, Reynolds number and Froude number based on the exit conditions:

4.1 $$\begin{array}{rl} & \gamma =\frac{V_{e}}{A_{e}^{3/2}},\;Re_{m}=\frac{V_{e}}{\nu t_{e}A_{e}^{1/2}}\;\mathrm{and}\\ & \;Fr_{m}=\frac{V_{e}}{A_{e}^{5/4}{\left(\Delta \rho g/\rho \right)}^{1/2}t_{e}}. \end{array}$$

Figure 1*b* shows a regime diagram with typical values of *γ* and *Re*_*m*_ plotted for coughs, breathing with and without masks and indicates how the flow patterns change depending on the type of expulsion. Through the mouth and nose, the geometrical parameter *γ* is typically large, but is greatly reduced when the mouth is enclosed by a mask. The Reynolds number indicates the potential for a turbulent initial jet. Masks play a role in reducing horizontal momentum and decreasing the overall Reynolds number, which leads to a greater dominance of buoyancy effects.

### 4.2. Flow development

Figure 1*c* shows a typical development of the expulsion of a vapour cloud from the mouth and nose along with a single image that highlights the eddy structure formed late after expulsion. The exhaled air forms an initially turbulent jet that entrains ambient air (diffuse lighting suppressed to some extend the small structures suggesting unduly a smooth front). The special characteristic of the flow is that its velocity decreases rapidly with distance from the point of ejection. Exhalation from the nose creates two jets that combine around the level of the chin and strongly interact with the torso. This interaction leads to an inhibition of entrainment and the creation of secondary vorticity that greatly widens the jet.

The expulsion characteristics are analysed using a standard computational framework (see appendix A) to assess the influence of the injection period and orifice shape on the exhaled cloud. Expulsion was modelled as a uniform flow, speed *u*_0_, applied normal to an elliptical orifice for a period *t*_*e*_. To follow the expelled air, an Eulerian approach was applied where a passive tracer (with the same diffusivity as *ν*) was introduced at the exit plane. Additional information was obtained from a Lagrangian approach by following particles of *d* = 1 μm in diameter (moving according to (3.2)) and injected at a rate of 10 ks^−1^ in time (figure 3*c*).

The physics of injection have been comprehensively studied in the range *γ* ∼ 2–5, *Re*_*m*_ = 1000–5000 in the context of vortex generation and so it is worth making a few remarks to put pneumodynamics into context. Vortex impulse has been a running theme for vortex generation and propagation for at least 40 years. Osborne Reynolds pointed out the loss of mass (or detrainment) from turbulent vortices, which Maxworthy [29,30] recasted as being a loss of vortex impulse over time. It was apparent that the movement and growth of a turbulent vortex could not be explained by invoking conservation of vortex impulse. Here, the global conservation of impulse is not in doubt—it is the partition between the impulse shared between the vortex and the ambient. It would seem appropriate to therefore question whether the impulse of the eddy is conserved [31] as it forms the basis of most models of cough dynamics [10,18].

**Figure 2.**
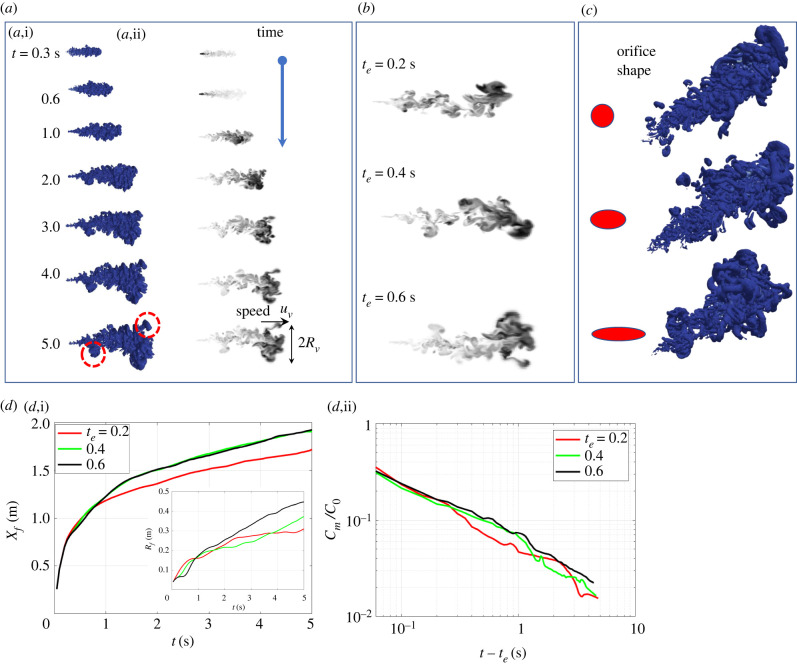
(*a*) Time sequence of a cough event created by flow through a circular orifice (*R*_0_ = 1 cm) with a speed *u*_0_ = 10 m s^−1^ for a period of *t*_*e*_ = 0.6 s. (*a*,i) Iso-contour of the second invariant of velocity gradient tensor *II* = 0.005 s^−2^, highlighting vortical regions of the flow; (*a*,ii) scalar concentration in a vertical slice in the flow; the intensity is normalized on the maximum value in the plane. The red dashed circles indicate intense vortices that are ejected from the jet. (*b*) Comparison between the state of the flow at *t* = 5 s for three injection times (*t*_*e*_ = 0.2, 0.4 and 0.6 s). (*c*) Effect of orifice shape, comparing elliptical orifices with aspect ratio 1, 2 and 4, but with identical area. A snap shot of the iso-contour of *II* = 0.005 s^−2^ is shown at *t* = 4 s. (*d*,i) Characteristics of the maximum displacement and width *X*_*f*_ and *R*_*f*_ (insert) as a function of time, and (*d*,ii) the decrease in the maximum concentration *C*_*m*_, after the injection period (*C*_0_ = 1).

Figure 2*a* shows the evolution of the flow as an isocontour of *II* (the second invariant of the velocity gradient tensor) which acts as a discriminator for the presence of vorticity, during the injection period (0 < *t* < *t*_*e*_ = 0.6 s) and in the period when the expulsion has reached a distance of 1.5–2 m. A passive scalar is followed in time and its distribution in a plane through the flow is shown. Since the maximum concentration decreases rapidly, the intensity is rescaled at each time. The three-dimensional image shows an isolated vortex expelled from the jet core (indicated by the red circles in figure 2*a*). This well-known feature was discussed by Scorer—its impact on infection transport is negligible because its volume is small and the concentration is greatly diminished by other physical processes.

The initial injection phase is analysed using a steady turbulent jet whose width grows due to entrainment that corresponds to a net flux across the jet interface proportional to the mean jet velocity [32]. The entrainment coefficient for a circular jet under steady conditions is *α*, so that the jet radius is *R* = *R*_0_ + 2*αx*, and the velocity of the jet is *u*/*u*_0_ = *R*_0_/*R*. The speed of the jet front propagates according to *X*_*f*_ = *R*_0_(*u*_0_ *t*/*αR*_0_)^1/2^. Ejection is completed at a time *t*_*e*_ = *V*_*e*_/*A*_*e*_ *u*_0_ at which stage the front is at a distance *X*_*f*_ from the source and moving with a speed *u*_*f*_ = (1/2)(*u*_0_ *R*_0_/*αt*_*e*_)^1/2^. After injection, the forward movement is faster for shorter injection periods, as confirmed in figure 2*d*. The volume flux in the jet increases with distance from the point of injection—at its termination and prior to the transformation into an eddy structure, the volume flux is estimated to be *Q*_*w*_ ∼ *u*_0_ *A*_*e*_ (*αX*_*f*_/*R*_0_).

The adjustment process for *γ* ≫ 1 from a turbulent jet to a propagating eddy is quite different from the case of *γ* ∼ 1 because the flow is characterized by ∂*u*/∂*s* < 0. This means that immediately after injection has ceased (*t* > *t*_*e*_), the self-induced motion leads to the wake of the vortex catching up to its front. A relatively crude approach is to ascribe the increase of the vortex impulse and volume from the jet to be

4.2 $$\frac{d}{dt}((1+C_{m})u_{v}V_{v})=u_{0}^{2}A_{e}\;\mathrm{and}\;\frac{dV_{v}}{dt}=Q_{w},$$

where *C*_*m*_ = 1/2 is the added-mass coefficient typical of a spherical vortex. The simple solution yields expressions for the vortex speed *u*_*v*_ and radius *R*_*v*_:

4.3 $$u_{v}=\frac{(2/3)\pi u_{0}^{2}A_{e}t+u_{f}V_{i}}{Q_{w}t+V_{i}}∼\frac{2}{3}\frac{\pi u_{0}^{2}A_{e}}{Q_{w}}\;\mathrm{and}\;R_{v}∼{\left(\frac{3Q_{w}t}{4\pi }\right)}^{1/3},$$

where *V*_*i*_ is an approximate initial volume of the vortex ($V_{i}∼4\pi R_{f}^{3}/3$). As the vortex moves forward, the build up of fluid impulse is ultimately halted. This mode of development is quite different from the pure entrainment and constant impulse model that is currently used to described expulsion [10,18]. Fitting a power-law to *R*_*v*_ ∼ *t*^*a*^, shows that *a* ∼ 0.33 for *t*_*e*_ = 0.2 s with a slightly faster increase for the longer injection (*t*_*e*_ = 0.6 s) where *a* ∼ 0.5. The numerical results tend to show a vortex speed that is approximately constant and independent of *t*_*e*_. The simple model captures some of the features of the vortex adjustment phase and is a useful starting point to understanding vortex formation for *γ* ≫ 1.

Orifice shape comes into play in the near field through the vortex dynamics and jet instability, which are not captured by classical jet models. Figure 2*c* shows a comparison between circular and elliptical orifices and confirms that circular orifices produce narrow jets that penetrate further than equivalent elliptical jets. The flow eccentricity leads to elliptical vortices that deform out of plane due to high curvature edges having a faster self-induced velocity than the low curvature elements. This leads to a more rapid progression to jet break-up, which reduces the jet penetration distance.

### 4.3. Mixing and dispersal due to breathing

The expelled air can be distinguished from the ambient in figure 2*a* by the presence of the scalar and the vorticity, since the ambient air is initially deficient in both. The complicated corrugated edge or interface between the inner (turbulent) and outer (non-turbulent) region—the TNTI—is a flow structure with complex and interesting physics [33,34]. The characteristics of TNTIs are not universal. Entrainment during the jet formation is largely through an engulfment mechanism where the interfaces are typically characterized by an enhancement in vorticity magnitude due to continuous stretching. Despite the diffusivity of passive material (e.g. particle concentration, temperature, humidity) being small, the engulfment process strains and shears entrained clean air which quickly mixes and dilutes the expelled air. This local mixing process occurs on a scale ${\left(DL/u_{*}\right)}^{1/2}$, where the integral scale of the turbulence is *L* and velocity scale $u_{*}$. This shows that the mixing of passive material and momentum tend to be quite similar even though both have quite different dynamical roles.

After the injection process has ceased, the leading eddy grows through a process that is typically described as entrainment. Entrainment occurs through the front of the eddy, but this tends to be much smaller than the inviscid flux through the rear of the vortex. The impulse of the vortex grows due to the mean momentum flux carried by the jet. The turbulent intensity (as measured by *u*_RMS_/*u*) within this region is typically high, with a weak mean flow responsible for the movement of the vortex. During this later stage, the maximum velocity shifts from the injection point to within the eddy and the concentration of the scalar decreases rapidly. After the injection processes has ceased, the maximum scalar concentration appears to decrease as (*t* − *t*_*e*_)^−3/2^ (figure 2*d*(ii)).

## 5. Influence of environmental factors on dispersal

### 5.1. Influence of ambient turbulence

Turbulence is an essential component of mechanical ventilation and is used to mix air and dilute contaminants, prior to air removal. While turbulence is a critical feature of the local environment, its effect on the spreading of expelled air has not been previously studied. Here we analyse the expulsion of air into a homogeneous turbulent flow characterized by an integral *L* and RMS velocity *u*_RMS_. The RMS velocity typically varies over the range *u*_RMS_ = 0.02–0.2 m s^−1^. The integral scale increases with distance from a diffuser, at around head level, it would be in the range 0.2–1.0 m.

The main characteristics of a jet moving into a turbulent region are discussed by Hunt [35] and explored through a series of experiments by Askin *et al*. [36] and Khorsandi *et al*. [37]. The main flow features are that for *u* ∼ *u*_RMS_ entrainment is suppressed and the boundary velocity increases dramatically, causing rapid dilution. Arnold *et al*. [38] analysed the interaction between a vortex and turbulence. As with other more recent studies, it appears that a rather crucial part of later stage development depends on the external turbulence disrupting the vortex through a significant modification of the rear wake and through a more complex modification of the turbulence around the vortex. The later disruption and spreading is generally through a non-Fickian or ballastic spread that occurs on scales up to, and comparable to, the integral scale of the ambient turbulence.

**Figure 3.**
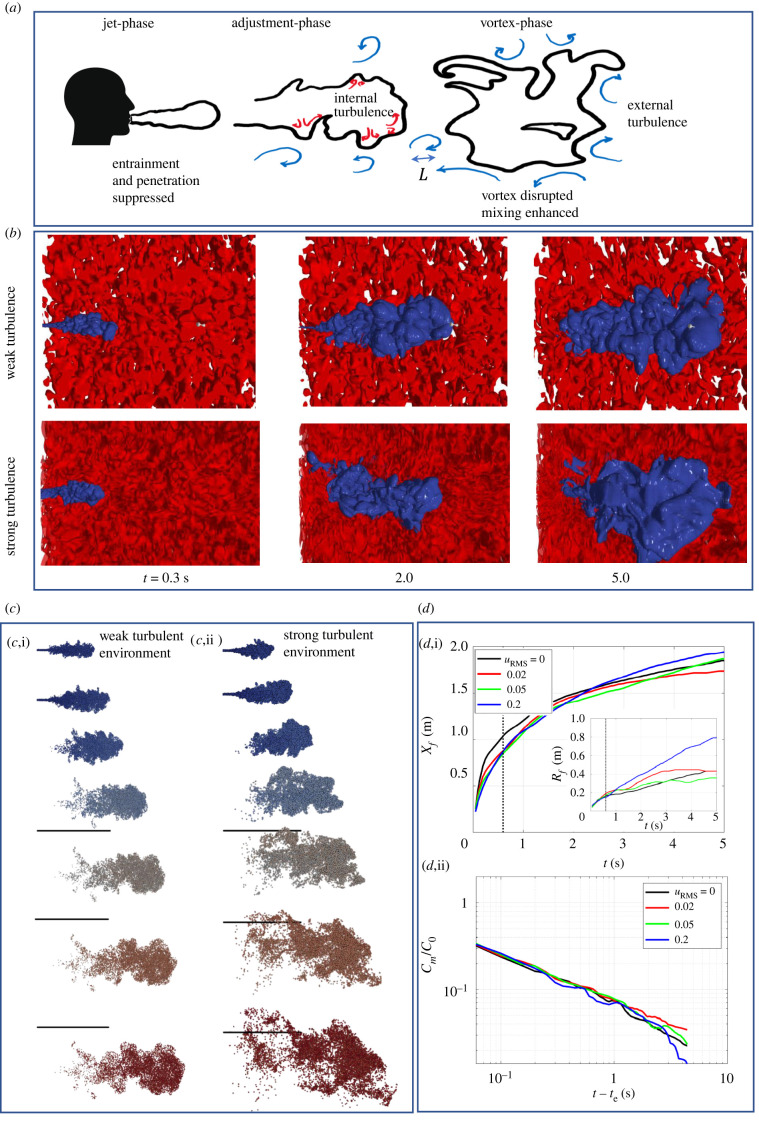
(*a*) Schematic showing the main phases of the development of exhaled air in the presence of external turbulence with rms velocity *u*_RMS_ and integral scale *L*. This consists of the jet phase, adjustment phase and the vortex phase. (*b*) Flow development for low and high levels of turbulence with weak and strong turbulence, with *u*_RMS_ = 0.02 and 0.2 m s^−1^ respectively. Here *u*_0_ = 10 m s^−1^, *t*_*e*_ = 0.6 s, *L* = 0.2 m. (*c*) Distribution and spreading of 1 μm particles, injected into the flow during the period *t* < *t*_*e*_ (Lagrangian simulation). The snap shots show the consequence of weak and strong turbulence at times *t* = 0.3, 0.6, 1, 2, 3, 4 and 5 s. The black line indicates a distance of 1 m. (*d*,i) Characteristics of the maximum displacement and width *X*_*f*_ and *R*_*f*_ (insert) of the jet as a function of time, and (*d*,ii) decrease in maximum concentration *C*_*m*_ after the injection period (with *C*_0_ = 1).

Figure 3 shows the consequence of exhalation into a homogeneous turbulent flow. A schematic of the main processes is shown in figure 3*a*, indicating the effect in the near field during the jet evolution, the adjustment period and the effect on the vortex phase. Figure 3*b* shows the isocontour of the passive scalar along with a cut through the flow with an isocontour of *II* = 0.005 s^−2^ to highlight the external flow. Contrasting the low turbulence case (*u*_RMS_ = 0.05 m s^−1^) with the high turbulence case (*u*_RMS_ = 0.2 m s^−1^) shows how the expulsion eddy is completely disrupted with the large-scale undulations on the interface on the approximate scale as *L* (figure 3*a*). The propagating eddy plays a role in distorting the ambient turbulence leading to a permanent transfer of the eddy impulse to the ambient. Figure 3*c* shows the spatial distribution of 1 μm particles injected with the expelled air. The particles are small enough that they move passively with the flow and are indicative of the movement of aerosol particles. External turbulence inhibits vortex penetration and during the later stage the enhanced lateral and longitudinal spread is clear.

Typically during the jet formation, the influence of external turbulence tends to be weak because the injection velocity is large compared to *u*_RMS_. The influences occurs during the eddy-vortex adjustment period when the mean flow becomes comparable to *u*_RMS_. As a consequence of the integral scale being larger than the jet source, the dispersion tends to be ballistic so that $R\approx R_{0}+u_{*}t$ (which is consistent with observations in figure 3*d*). The disruption of the vortex means that its propagation moves from self-induced motion to being shredded by the ambient turbulence. This rapid process leads to the increased spread by greater dilution of the expelled air and the penetration halted.

### 5.2. Influence of ambient temperature and RH

The difference between the constituent properties (temperature and RH) of the expelled and ambient air, affects both the dynamics of the flow and the long-term fate of droplets. Temperature and RH both decrease air density and through the action of a buoyancy force, lead to air rising. As indicated by (4.1), the Froude number for expulsion is typically very high (except, for instance, a loosely fitting mask) and consequently buoyancy forces are typically weak during the injection period. As the flow slows down, the local Reynolds number decreases, entrainment is suppressed and buoyancy may become dominant causing the vortex to rise.

**Figure 4.**
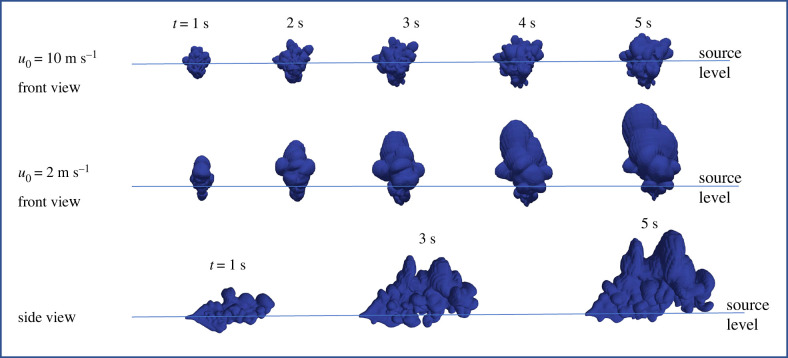
Sequence showing the influence of buoyancy contrast Δ*ρ*/*ρ*_0_ = −0.1 on the development of expelled air through a circular orifice *R*_0_ = 1 cm over a period *t*_*e*_ = 0.6 s. The buoyancy contrast occurs through a density difference between the expelled air and the uniform ambient (of density *ρ*_0_). The isocontour of a scalar introduced into the jet is shown at a level *C* = 0.001. A comparison is shown between a high-speed cough (*u*_0_ = 10 m s^−1^) and breathing (*u*_0_ = 2 m s^−1^). Front and side views are shown for comparison. The horizontal lines indicate the level of expulsion.

These processes are illustrated computationally in figure 4 where the density contrast is linked to the presence of a passive scalar *C* through Δ*ρ* = *C*Δ*ρ*_0_, whose evolution in time is followed and coupled to the momentum equation. At fixed pressure, the fractional density difference can be interpreted in terms of a temperature differential Δ*T* through Δ*ρ* = *ρ*_0_Δ*T*/*T*_0_. Figure 4 shows a comparison between the evolution of a buoyant jet ejected at low and high speeds. For slowly moving jets, buoyancy influences the flow leading to a clear rise of the expelled air over the short simulation period. The practical significance is that the penetration distance of expulsion will be arrested by thermal effects with stratification ultimately limiting the rise height. In general, the transport processes and dynamics are not captured by integral models because the eddies in the jet are comparable to the integral scale in the ambient turbulence.

## 6. Room scale transport

The fundamental processes by which indoor air mixes—through mechanical or natural ventilation—are largely controlled by turbulence and air density respectively, with their spatial variation greatly affecting the efficiency of mixing and flushing. The generation of turbulence and reinforcement of stratification continuously changes due to people movement, equipment, doors opening/closing and transient heating. In this section, we discuss some of the critical physics.

### 6.1. Mechanical ventilation

**Figure 5.**
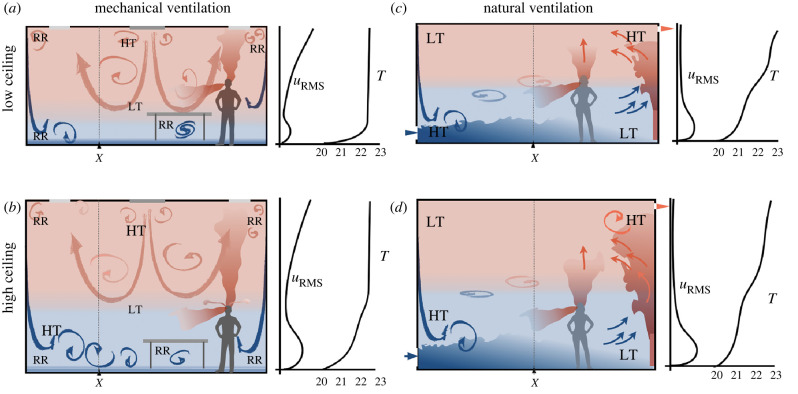
Schematics contrasting the air flow and thermal stratification seen during mechanical (*a*,*b*) and natural ventilation (*c*,*d*) in a room, highlighting how the processes change as the room height increases from *h* = 2.7 m (*a*,*c*) to *h* = 5 m (*b*,*d*). The flow generates high turbulence (HT) regions, recirculating regions (RR) and regions of low turbulence (LT). The thermal plume created by heating near the wall generates turbulence and an entrainment field. The continuous volume flux of dense cold air into the room is balanced by a flux of hot air through a vent, usually located near the ceiling. The mechanical ventilation is generated by a diffuser and an outlet—both typically located at the ceiling level. An indication of the typical vertical variation of RMS velocity (*u*_RMS_) and temperature (*T*) is drawn for each sketch at position *X*.

Mechanical ventilation uses high levels of turbulence to promote mixing/dilution and disrupt thermal stratification, with a mean flow to displace or flush out well-mixed air. The flow of clean or filtered air is typically characterized by *u*_RMS_/*u* ∼ 1 near the inlet or diffuser. Through entrainment, *u*_RMS_ decreases rather rapidly (over a scale comparable to the diffuser blades) and the integral scale increases (figure 5*a*). To disrupt thermal stratification and to ensure the comfort of people beneath, the diffuser generally spreads air laterally and with the Coanda effect, hugs the ceiling. The mean flow decreases as 1/*r* from the diffuser (*r* being the distance from the outlet) since the flow is usually confined in one direction. Air is removed from the room via an outlet, placed on the ceiling or high on the wall. Air removal creates an irrotational sink flow that decays much faster than 1/*r*^2^ and the flow has a very weak dependence on shape when compact. To increase their effectiveness, many offices and hospitals use long narrow inlets which means that their flow field decays at a rate closer to 1/*r*. The purpose is to mechanically mix the air and temperature stratification if present, and to refresh it with an exchange flow.

Mechanical ventilation systems are usually sized on ideas around perfect mixing. When clean air is introduced into a room with volume *V* at a volume flux *Q*, the mean concentration of an airborne contaminant that occupies a room decays *C*_*T*_ = exp (−*Qt*/*V*). The initial decrease of *C*_*T*_ is due to displacement flushing, caused by mechanical ventilation, while the exponential decrease is due to mixing and flushing. These estimates are actually quite close to idealized tests in closed rooms, with little furniture and adiabatic walls [39]. The ratio *Q*/*V* is typically described as the air-changes per hour (ACH, when *Q* is cast in m^3^ h^−1^) and is a suitable metric for the effectiveness of a ventilation system. For surgery and delivery rooms, ACH = 15, with ACH varying in different spaces, for example, a requirement to have ACH ∼ 10 for rooms with AGP procedures during dentistry. The presence of furniture and people introduces regions of long residence time created by recirculating regions (figure 5*a*), which causes a significant deviation from the exponential decay law for *C*_*T*_. The most challenging aspect is that the flow induced by the ventilation system can be fundamentally altered by doors opening events and buoyancy-driven exchange flows.

### 6.2. Natural ventilation

Natural ventilation is a general description of passive methods of driving an exchange flow between internal and external spaces. The two primary mechanisms are through the use of buoyancy and wind—with the latter generating a pressure differential between openings [40]. When thermal effects generate a buoyancy-driven exchange flow between air inside the room and clean air from outside the room, the air in the room becomes stratified, with the hot air flowing out near the top and by continuity, cool air entering at floor level. The basic principle is that stratification separates warm contaminated air from cooler fresh clean air. Ideally, with window openings, the temperature interface should be above head height, with the cooler air in the lower layer being well mixed so that exhaled air rises to the upper warm air layer (figure 4), where it is continuously removed [40]. The weak turbulence in the lower layer causes fine droplets to sediment more quickly, with the aerosol rising in the warm breath entering the upper layer and escaping through the outlet. Turbulence due to convection or wind results in fast mixing and displacement (e.g. [41]). Note that exhaled air due to its relatively high temperature and nearby body heat generates an upward plume motion—as mostly shown in the literature—but that the momentum of a cough is downward or horizontal as shown in figure 1 [see 42] (see figure 5). The dispersion of droplets are affected by the temperature stratification and humidity, and when turned into aerosols, follow the routes set by roomscale circulation.

In most indoor spaces, the air is warmed up by heaters at the wall and fresh air passes along the floor. Heat transfer between the walls, floor and ambient air contributes to the establishment of a stable temperature stratification (see figure 5*c*,*d*). This stratification continuously evolves over a day due to varying thermal loads (transient heating, thermal inertia of rooms which cool during the evening and weekends) and the exchange flows enhanced by doors that open and close. Thermal comfort is sometimes controlled using fan heaters (in cold weather) or ceiling mounted fans (in hot weather) which assist in disrupting thermal stratification and mixing room air. While the highest ACH with natural ventilation occurs in the presence of wind, it is generally not used because it creates challenges for thermal comfort.

In hotter climates, natural ventilation tends to be promoted through the use of air-conditioning that provides a source of cooler air that flows along the ground and hot air entering through doors and windows. The environmental cost in maintaining natural ventilation may be significant since it requires heat (or cooling) to promote a ventilation exchange flow.

In view of viral air contamination, the main benefit of natural ventilation is that the level of turbulence is low *u*_RMS_/*u* ≪ 1 so that droplets have a strong tendency to sediment to the ground, and the potential for trapping in circulation regions is diminished. Stratification however confines exhaled aerosols at the height where they are emitted.

### 6.3. Influence of room height

As the room height increases, the potential for mechanical ventilation to disrupt the thermal stratification decreases due to the increased distance between the inlet and the ground (figure 5*b*). Mechanical ventilation is therefore not ideal for high ceilings, and limitations are often addressed by lowering the height of the diffusers.

The heat exchange via walls and floors are often ignored in ventilation studies and are treated as adiabatic. A temperature difference of only 1°C at the wall will generate a turbulent wall plume at a height less than a metre, since the Grashof number, *Gr* = *g*Δ*T h*^3^/*T*_0_*ν*^2^, which predicts the onset of turbulence, is typically greater than the threshold value of 10^9^ (where *h* the room height, *T*_0_ is the room temperature). The taller the room, the higher the turbulence intensity. In the absence of ventilation, this wall cooling leads to a linear stratification in the cavity adjacent to it (e.g. [43]). The generation of a thermal stratification, especially in large spaces, leads to poor mixing and high risk of COVID transmission through the air. In mechanically ventilated spaces, even for quite high ACH, small temperature differences between the air and walls can promote vertical buoyancy-driven wall-bound flows [5].

Natural ventilation is much more effective in rooms with high ceilings because the buoyancy force is larger (figure 5*d*). For balanced flows with low or high ceilings, weak (linear) stratification of less than 1°C in the ambient temperature (at 20°C) can cause the exhaled air jet to collapses into a relatively thin layer around the height of its emission [44]. The exhaled breath with the small aerosols remain at this height. For unbalanced flows with high ceilings and large temperature differences, there is a greater tendency to create thermal stratification with an interface height that lies above the head of people, and which causes warm exhaled breath to rise and reduce the risk to transmission between people in close proximity. Therefore, mixing of the lower layer as well as its refreshment with outside air are crucial for having optimal air conditions with low contamination risks.

## 7. Concluding remarks

Mitigating the airborne transmission of disease in enclosed spaces requires understanding key fluid mechanical processes associated with the movement of infectious agents, specifically the influence of turbulence, buoyancy and stratification. The complexity of this challenge comes from the spatial variation of turbulence and stratification, which in a real environment, occurs due to the movement of people, heat transfer processes, ventilation and the impact of the local climate.

In this paper we have discussed pneumodynamics and shown how more fundamental concepts need to be revisited to explain how coherent structures develop and are strongly affected by external turbulence and buoyancy on dispersion. These processes form a connection between local transport mediated by coughing and breathing, and ventilation that transforms it into long-range effects and risks. COVID-19 has provided an opportunity to look once again at the fundamental processes of the spread of infectious diseases. This has provided a fresh opportunity to examine established orthodoxies, and to develop new conceptual frameworks whose impact lies beyond infection control. Part of this challenge will have to be met by the turbulence community, especially in understanding transport processes in a lived environment.

## Data accessibility

All the computational data was generated using open-source software (https://openfoam.org/).

## Appendix A

The numerical simulations were undertaken, using a general open-source solver—OpenFOAM v1806. The standard pimpleFoam solver was supplemented with a solver for a passive scalar along with an LPT model, modified to follow aerosol particles whose diameter was fixed at *d* = 1 μm. The turbulence model option chosen was Spalart-Allmaras DDES. The source flow was injected with a constant flux over a prescribed area.

A highly structured mesh was applied to solve the problem, that formed a cylindrical control volume (of radius 1.5 m, length 3 m) that conformed onto an elliptical inlet section, through an intermediate circular mesh of radius 10 cm. Angular discretization was fixed at $N_{\theta }=96$, while the radial and longitudinal discretizations were in total 250 and 500 elements. The mesh size was varied spatially with a total of 4.5 M cells.
